# Supplementary material for: Glacial dysoxia in the deep subpolar North Atlantic during the Mid-Pleistocene Transition
Source: Nat Commun. 2026 Apr 24;17:3748. doi: 10.1038/s41467-026-71268-4 (PMC13109349; doi:10.1038/s41467-026-71268-4)
Supplement: Supplementary file 1 — Supplementary information [file 41467_2026_71268_MOESM1_ESM.pdf]

Supplementary information

Sedimentary environments at Site U1314 and 1094

Site U1314 and 1094 have comparable sedimentation rates during the Mid-Pleistocene Transition (MPT; 1100–780 kyr) of ca. 9 cm/kyr [1, 2]. Ca/Fe is usually interpreted as a proxy for detrital versus biogenic input [3]. Comparison of Ca/Fe values from 1094 [XRF; 3] and U1314 (this study) shows that, except the short-lived peaks during the interglacial maxima at Site 1094, the values are in the similar range (Supplementary Fig. 1). Moreover, detrital input, as expressed in lithic grains per gram, is very similar in both sites, with values between 2–8 $\times 10^3$ grains/g [1, 4]. Since sedimentation and detrital input in both sites are very similar, these factors are discarded as having a potential factor in burial conditions and/or remobilization that could lead to different diagenesis conditions which could affect redox conditions at both sites.

Manganese in bulk sediments and in planktonic foraminifera coatings

Mn-rich phases in bulk sediments and associated with foraminifera tests precipitated as Mn-oxides (the term "Mn-oxide" is used interchangeably with "Mn-Fe-oxide" here) and Mn-rich carbonates when oxygen-rich conditions are encountered [5-9]. The behavior of Mn depends on porewater redox conditions. Under the presence of O_2 at the sediment surface, authigenic solid phase Mn accumulates as MnO_2 coatings (as Mn^{3+} and Mn^{4+}), whereas burial under reducing conditions do not favor authigenic Mn precipitation, and instead Mn remains soluble as Mn^{2+} in porewaters [see 5 and references therein]. Under oxygen-rich conditions, Mn is then mainly removed from porewater by the formation of Mn-rich carbonates and/or oxides (although this is not an exclusive removal mechanism), mainly at the inner surface of foraminifera tests due the reactive surfaces that promote nucleation [10]. Identification of Mn-rich authigenic phases on foraminiferal tests requires the use of one or several analytical techniques (LA-ICP-MS, ICP-MS, XRD, SEM, EDX) [6]. However, because Mn oxides can be removed through a reductive cleaning step, whereas authigenic Mn carbonates persist after standard solution-based cleaning methods and even with reductive cleaning step [11], we suggest that the authigenic Mn present in planktonic foraminifera at both Sites U1314 and 1094 are Mn-rich carbonates. In addition, studies including samples from multiple ocean regions found observed that values >200 µmol/mol appears to be related to diagenetic Mn-carbonate coatings on the foraminiferal shells [12].
Authigenic Mn maxima in sediments and foraminifera are found in the Pacific Southern Ocean and Equatorial Pacific, mainly during Middle-to-Late Pleistocene deglaciations [13] (Supplementary Fig. 1). Dissolved Mn can diffuse up core, above the depth of complete O_2 consumption, where it reprecipitates as solid Mn. Therefore, the authigenic Mn maxima represented the depth immediately above O_2 depleted conditions [14]. Organic carbon export to the seafloor, sedimentation rate and bottom oxygen concentration are the drivers of the depth of this pore water redox front [7]. Decreasing the supply of organic matter to sediments reduces the rate at which oxygen is consumed by aerobic respiration in porewaters, thus allowing well-oxygenated porewater to persist to greater depths. Increasing bottom and pore water oxygen levels (BPWO) concentrations push the initial O_2 content of the porewaters higher, allowing porewater oxygen concentrations to reach greater depths for a given organic matter supply (see section "Ocean productivity and redox proxies").

The authigenic Mn peaks are formed a few centimeters below the sediment-water interface. This depth may vary from one location to another depending on (steady vs non-steady) diagenetic conditions and ambient burial conditions (e.g. sedimentation rate, bottom water oxygenation levels) [15]. A shallower oxygen penetration depth would shift the manganese (Mn) peak into more reducing pore waters, where Mn would dissolve and diffuse upwards, effectively removing any trace of the former peak. In contrast, if the oxygen penetration depth increases in the sediment, the Mn peak remains in oxygenated conditions and is thus preserved [16]. One advantage of using Mn/Ca ratios in foraminiferal calcite over bulk sediment proxies like Mn /Al is that, once Mn is incorporated into the foraminiferal shell, its concentration remains stable and is not affected by subsequent diagenetic redox processes [17, 18].

One important consideration is that the age of the observed Mn peak represents an upper limit for the initiation of changes in oxygenation, and the precise timing of the Mn peaks cannot be compared from one core to another. The timing of the deglacial increase in Mn (bulk sediment Mn/Al and Mn/Ca in planktonic foraminifera for Site U1314; Mn/Ca in planktonic foraminifera for Site 1094) are offset by 5-7 kyr, which could be due to either differences in burial conditions driven by variable accumulation rates between the two cores, or due to slight misalignment in wiggle matching the two age models. However, the timing of the Mn-precipitation within sediments deposited at the end of glacial periods in both sites is consistent with the $\delta^{18}O_b$ records at both sites and depicts timing of CO_2 release and the onset of well oxygenated bottom-water conditions in both subpolar regions. Moreover, the timing of the peaks in planktonic foraminifera Mn/Ca at Site 1094 [2] is also consistent with other proxies in the same core which are related to a change in the overturning in the Southern Ocean (Fig. 3), and consequently their stratigraphic position is not sensitive to ambient burial conditions. Therefore, although the leads and lags cannot be statistically quantified, and part of the offset could be partly attributed to age-model uncertainties or sedimentary conditions, we consider that part the lag in the Mn peak at Site U1314 relative to Site 1094 might represent a real delay of the re-initiation of the deep ocean ventilation in the North Atlantic relative to the Southern Ocean.

Timing of bulk Mn/Al and planktonic foraminifera Mn/Ca in both sites U1314 and 1094 indicates that, whatever the Mn-rich phase precipitating, either as Mn-rich authigenic minerals or foraminifera coatings, it is responding to BPWO levels (Supplementary Fig. 3A-B). While both sites were cleaned using similar protocols, Site U1314 does show peaks of Mn/Ca mainly during interglacial intervals, but also during glacials, compared to Site 1090, where peaks of Mn/Ca are only observed during interglacial periods. This difference in peak amplitude is partly due to the less-frequent dysoxic conditions at Site U1314, compared to the longer periods of dysoxia at Site 1094, but also due to the amount of initial Mn precipitated in the foraminifera shell [6, 19]. Correlation between authigenic and Mn/Ca in planktonic foraminifera coatings in Site 1094 is higher during interglacial and termination event sections of the record, because Mn contamination is much more prevalent during these intervals [2]. At Site 1094, Mn/Ca > 100-200 μmol/mol can be indicative of potential Mn-Fe coating material and the cleaning procedures do not fully remove these authigenic phases, which precipitate under oxic conditions [20], such as the peaks with Mn/Ca > 800 μmol/mol recorded during deglaciations at 950, 860 and 780 kyr ago (Fig. 3). Near-zero Mn/Ca values during glacial periods are due to the dysoxic conditions that prevent Mn precipitation but also by the more efficient Mn removal by the cleaning procedure due to the lower Mn contamination during these intervals [20] (Supplementary Fig. 1B). At Site 1094, there is a high correlation between benthic and planktonic foraminifera coatings

(Supplementary Fig. 1C), indicating that a background Mn/Ca concentration is a persistent feature in all biogenic components of the sediment.

Sedimentary phosphorus components

The phosphorus components of the sediments are influenced by different phosphorous pools in the ocean and redox state of the bottom waters [21, 22]. The average total phosphorus and reactive (i.e. biologically available) phosphorus is 1089 ± 40 and 720 ± 50 µg/g, respectively, which are higher than phosphorus concentrations reported for other regions in the central North Atlantic located in lower-productivity areas [23-25]. Detrital phosphorus mainly derives from igneous and metamorphic material [23]. This fraction increases during higher deposition of ice-rafted debris at U1314 (Fig. 2F; Supplementary Fig. 4A). In contrast, organic, authigenic and Fe-bound phosphorus phases, all of which are influenced by the BPWO conditions, decrease in sediments during these episodes. These findings suggest that during the freshwater perturbations during ice-rafted debris events, dysaerobic conditions in the sediments may have fostered the loss of dissolved phosphorus from the sediments to the water column [26, 27] (Supplementary Fig. 4), as inferred in other deep sites in the North and South Atlantic using benthic foraminifera Cd/Ca-based PO_4^3 estimates [23, 28-30] (Supplementary Fig. 7). Dissolution of solid P phases in the sediment and release to the water column can also occur under a high export productivity scenario with high oxygen consumption during carbon remineralization [31]. However, this is unlikely at Site U1314, since episodes of higher productivity during interglacials (e.g. MIS 25, MIS 21 or MIS 19) are characterized by higher burial of reactive P (Supplementary Fig. 4E). Phosphorus can be remobilized in sediments after deposition. The main consequence of diagenetic alterations of P involve redistribution between phases [sink switching; 25]. Once buried below the most reactive surface layers, the bulk P concentrations are largely locked in, although redistribution occurs of P phases cause a decrease in the fraction of organically-bound and surface-bound P and an increase in authigenically-bound P in the form of authigenic carbonate fluorapatite minerals with increasing age/depth [32]. However, there are no apparent trends with age/depth the authigenic P fraction at Site U1314 (Supplementary Fig. 4C). Additionally, if diagenesis did redistribute P from the organic or Fe-bound P into the authigenic phase, this would not affect to our interpretation since the total amount of originally reactive P would still be locked into the sedimentary record at Site U1314 that we are interpreting.

Ocean productivity and redox-sensitive geochemical proxies

A decrease in porewater oxygenation can be achieved either through an increase in the supply of organic matter to sediments, or through a decrease in BPWO concentrations. Higher supplies of organic matter to sediments might/may increase the rate at which oxygen is consumed by aerobic respiration in porewaters. Locations where authigenic Mn maxima in have been observed in sediments are characterized by small changes in export productivity at glacial-interglacial timescale in the eastern equatorial Pacific (EEP) [13], increases in export production during deglaciations in the Southern Ocean [33-35], or by increases in export production during interglacial periods (U1314, this study, Supplementary Fig. 5). During the MPT, ocean primary productivity (biogenic silica and calcium carbonate) and carbon export (organic carbon) proxies show higher values during warm climate intervals (mainly interglacials) (Supplementary Fig. 5) at Site U1314, or at terminations at Site 1094

[XRF Ba/Fe; 33], simultaneous with increased BPWO as indicated by the redox-sensitive geochemical proxies (Mn/Al, P/Al, planktonic foraminifera Mn/Ca), and vice versa during glacials. Therefore, deglacial bulk Mn maxima can thus only be uniquely attributed to increased BPWO concentrations in locations where export productivity (i.e., sedimentary organic matter supply) is either constant or increasing through the deglaciation.

Benthic carbon isotope versus redox records at U1314

During glaciations between 1100–780 kyr, Site U1314 $\delta^{13}C_b$ records lower values which correspond to a reduced ventilation [36, 37]. $\delta^{13}C$ of *Lobatula wuellerstorfi* has been widely used as a tracer of past changes in deep ocean ventilation. However, the $\delta 13C_b$ signal may be affected by additionally environmental processes, such as air-sea gas exchange [38], non-stationary water-mass end-members [39], carbonate ion concentrations [40], biological export productivity signals [41] and bias towards specific microenvironment or changes in microhabitat [42]. As such, it is unsurprising to observe disagreements between $\delta^{13}C_b$ and other proxies sensitive to deep circulation. However, there is a significant correlation between $\delta^{13}C_b$ and redox-sensitive geochemical proxies (bulk sediment P/Al and Mn/Al, planktonic foraminifera Mn/Ca; $r = 0.38–0.32$, $p<0.05$) and the well-ventilated benthic foraminifera assemblage ($r=0.36$, $p<0.05$) (Supplementary Fig. 6) which are not affected by the same caveats, indicating that at least the qualitative change in the oxic vs dysoxic conditions at Site U1314 across time is consistent among proxies.

Taxonomic remarks

The taxonomic attributions of the species *Astrononion novozealandicum* Cushman and Edwards, 1937, *Astrononion echolsi* Kennett, 1967 and *Astrononion pusillum* Hornibrook, 1961 might be confusing in the specialized literature, which somehow complicates the scarce ecological information regarding these species. The specimens identified in Site U1314 are considered to belong to the species *Astrononion novozealandicum* Cushman and Edwards, 1937 (AphiaID: 466463, see images, Supplementary Fig. 9). Here, we follow the original description of *A. novozealandicum* (Cushman and Edwards, 1937, and holotype: https://collections.nmnh.si.edu/search/paleo/?ark=ark:/65665/376c23b2adfd34912a126cbb021cba96a, last time accessed on 29 July 2024) and other authors scanning electron microscope images and taxonomic discussions [43-46] to distinguish *A. novozealandicum* from *A. echolsi*. We consider that assignations of *A. echolsi* (holotype: https://collections.nmnh.si.edu/search/paleo/?ark=ark:/65665/33f22c14edb964613ac70e81bf49530c4) should be restricted to individuals with secondary apertures closer to umbilical area [see illustrations in 47, 48-50]. Following this criteria, based exclusively on morphological traits, attributions and images of *Astrononion echolsi* of Corliss [51], Mead [52], Belanger and Berggren [53], Nomura [54], and *Astrononion pusillum* of Miller and Katz [55] could be assigned to *A. novozealandicum*.

Supplementary figures

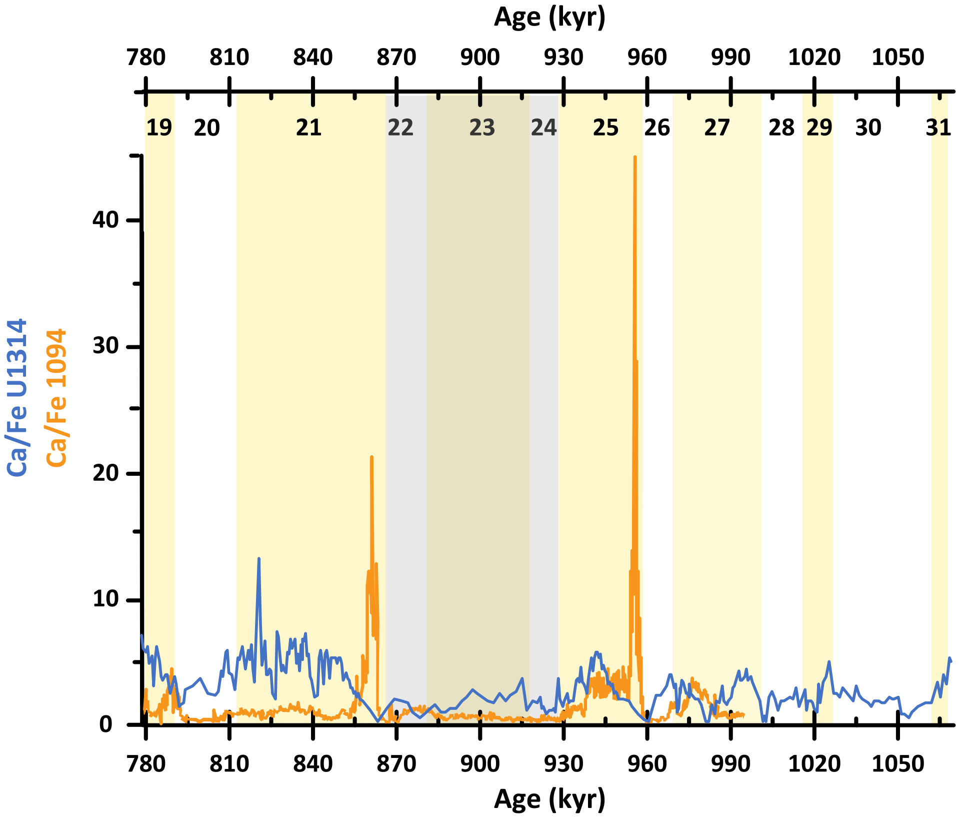

Supplementary Fig. 1. Ca/Fe ratios at Site U1314 (elemental geochemistry, this study) and 1094 (XRF) [3]. Black numbers indicate interglacials (odd) and glacials (even) marine isotope stages (MIS). Yellow vertical bars correspond to interglacial stages.

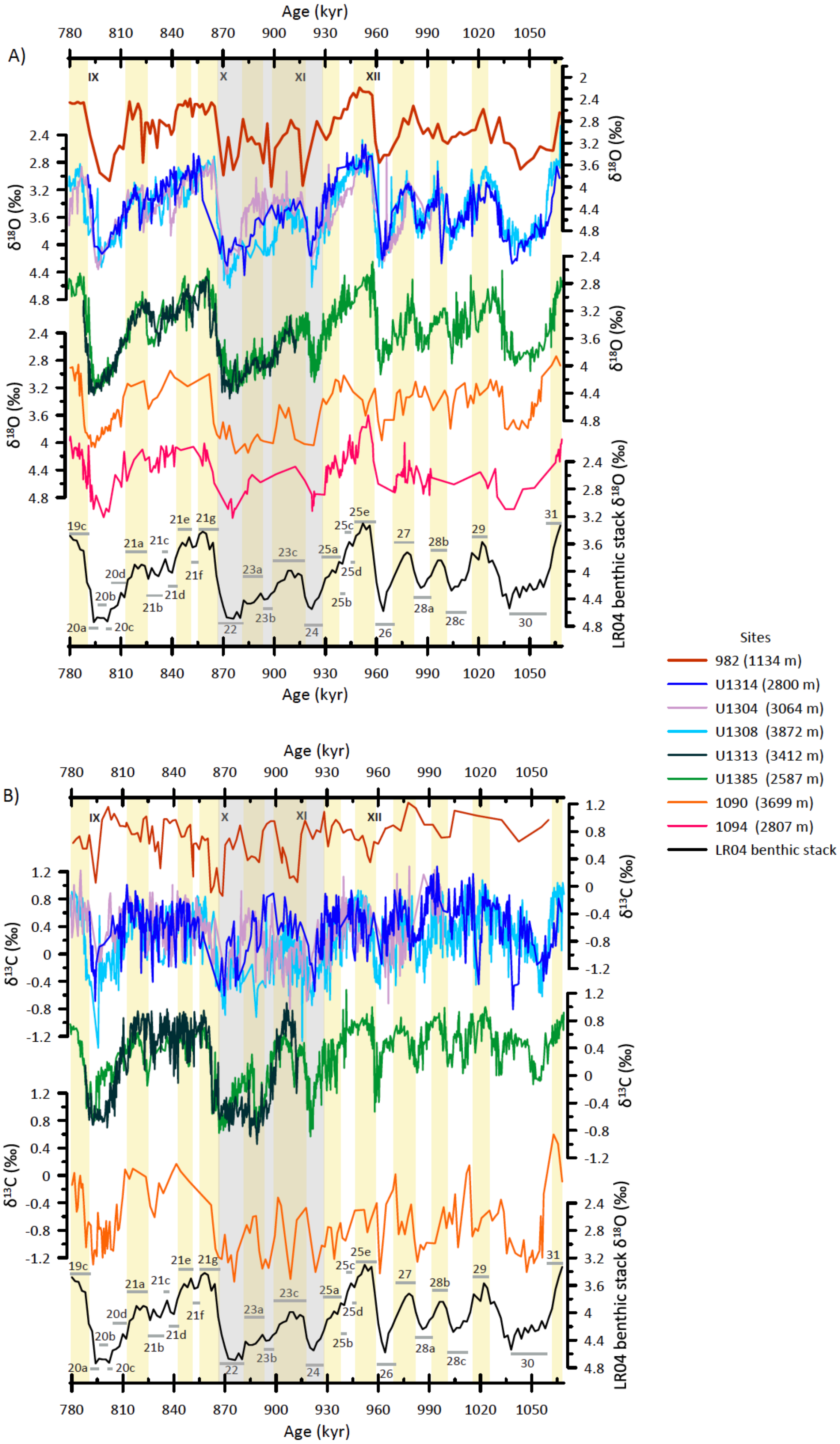

A)
B)
Age (kyr)
780
810
840
870
900
930
960
990
1020
1050
IX
X
XI
XII
δ18O (‰)
δ13C (‰)
LR04 benthic stack δ18O (‰)
2.4
2.8
3.2
3.6
4
4.4
4.8
1.2
0.8
0.4
0
-0.4
-0.8
-1.2
19c
20a
20b
20c
20d
21a
21b
21c
21d
21e
21f
21g
22
23a
23b
23c
24
25a
25b
25c
25d
25e
26
27
28a
28b
28c
29
30
31
Sites
982 (1134 m)
U1314 (2800 m)
U1304 (3064 m)
U1308 (3872 m)
U1313 (3412 m)
U1385 (2587 m)
1090 (3699 m)
1094 (2807 m)
LR04 benthic stack

Supplementary Fig. 2. Downcore stable isotope records in benthic foraminifera for different sites in the Atlantic basin [1, 2, 56-61] and the LR04 benthic stack [62]: (A) $\delta^{18}O$ and (B) $\delta^{13}C$. All records are plotted using their original timescale and selecting only *C. wuellerstorfi* or *Cibicidoides* spp. values (no correction applied). The vertical grey bar highlights MIS 24–MIS 22. Black numbers next to the LR04 benthic stack indicate interglacial/interstadial (yellow vertical bar) and glacial/stadial marine isotope stages (MIS), following the scheme by Railsback et al. [63]. Glacial termination events are noted with letters (IX-XII). Location of the cores is shown on figure 1 in the main manuscript.

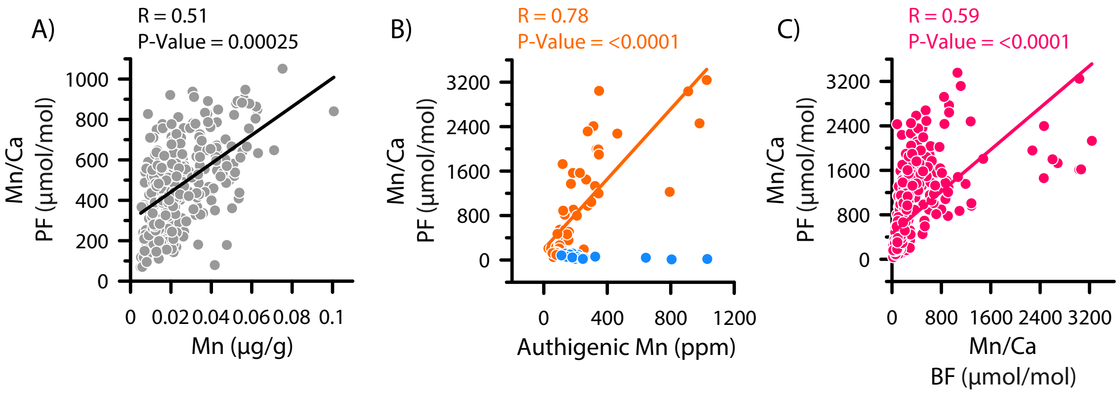

Supplementary Fig. 3. Correlation of Mn measured in different sediment components. (A) Correlation between Mn/Ca ratios from planktonic foraminifera coatings *(N. pachyderma*) and bulk Mn from IODP Site U1314 between MIS 31 and 19 [37]. (B) Same but for ODP Site 1094 during MIS 11 (orange dots) and 12 (blue dots) [2, 13]. (C) Correlation between Mn/Ca ratios from planktonic and benthic foraminifera (*Melonis pompilioides*) from ODP Site 1094 between 0.2 and 1500 ka [2].

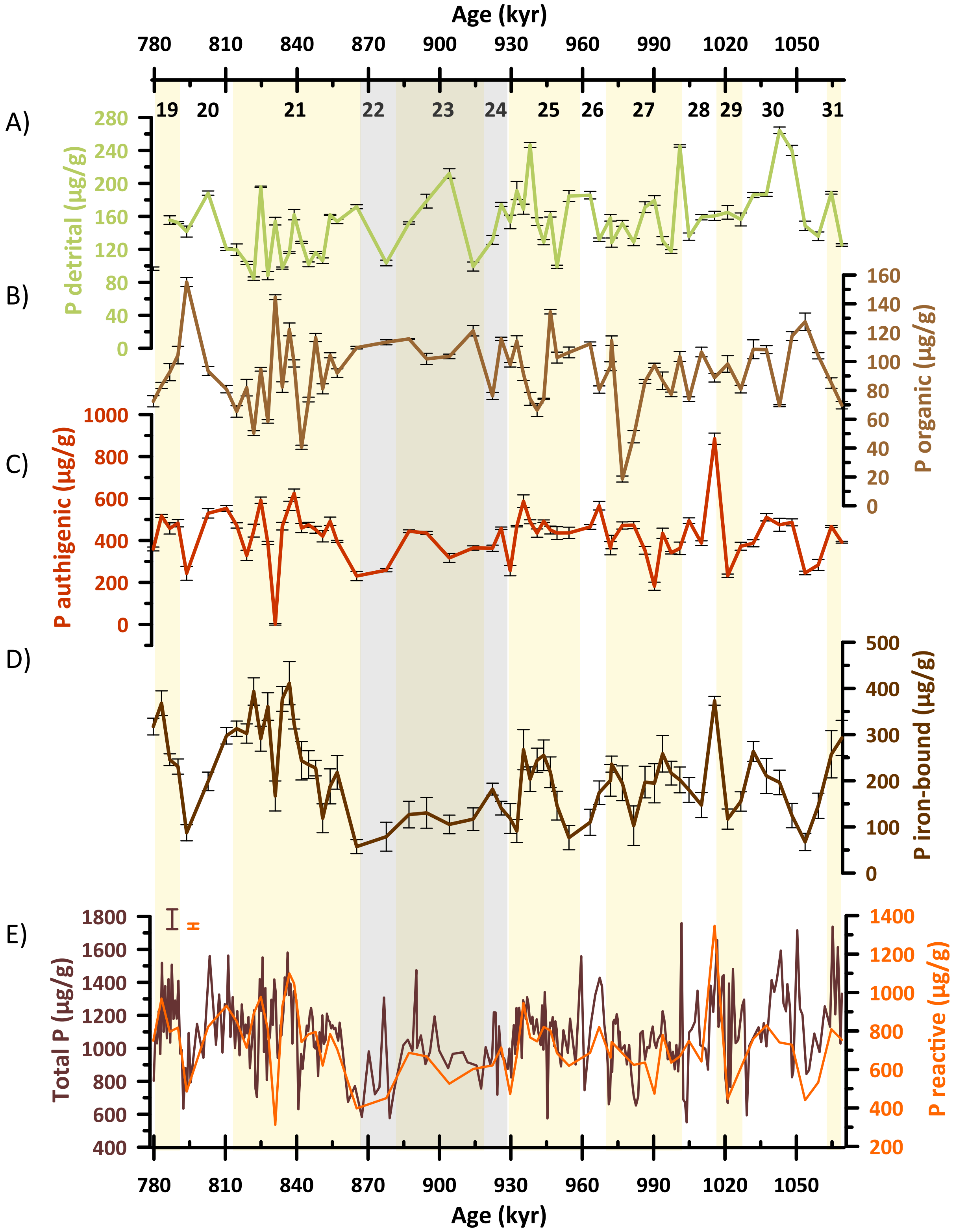

Supplementary Fig. 4. Downcore sedimentary phosphorus fractions at Site U1314: (A) detrital, (B) organic, (C) authigenic and (D) iron-bound. (E) Total phosphorus from elemental geochemistry (dark brown line) and phosphorus reactive (sum of all fractions, orange line). Error bars illustrate variation of reproducibility (1 SD) (Methods). Black numbers indicate interglacials (odd) and glacials (even) marine isotope stages (MIS). Yellow vertical bars correspond to interglacial stages.

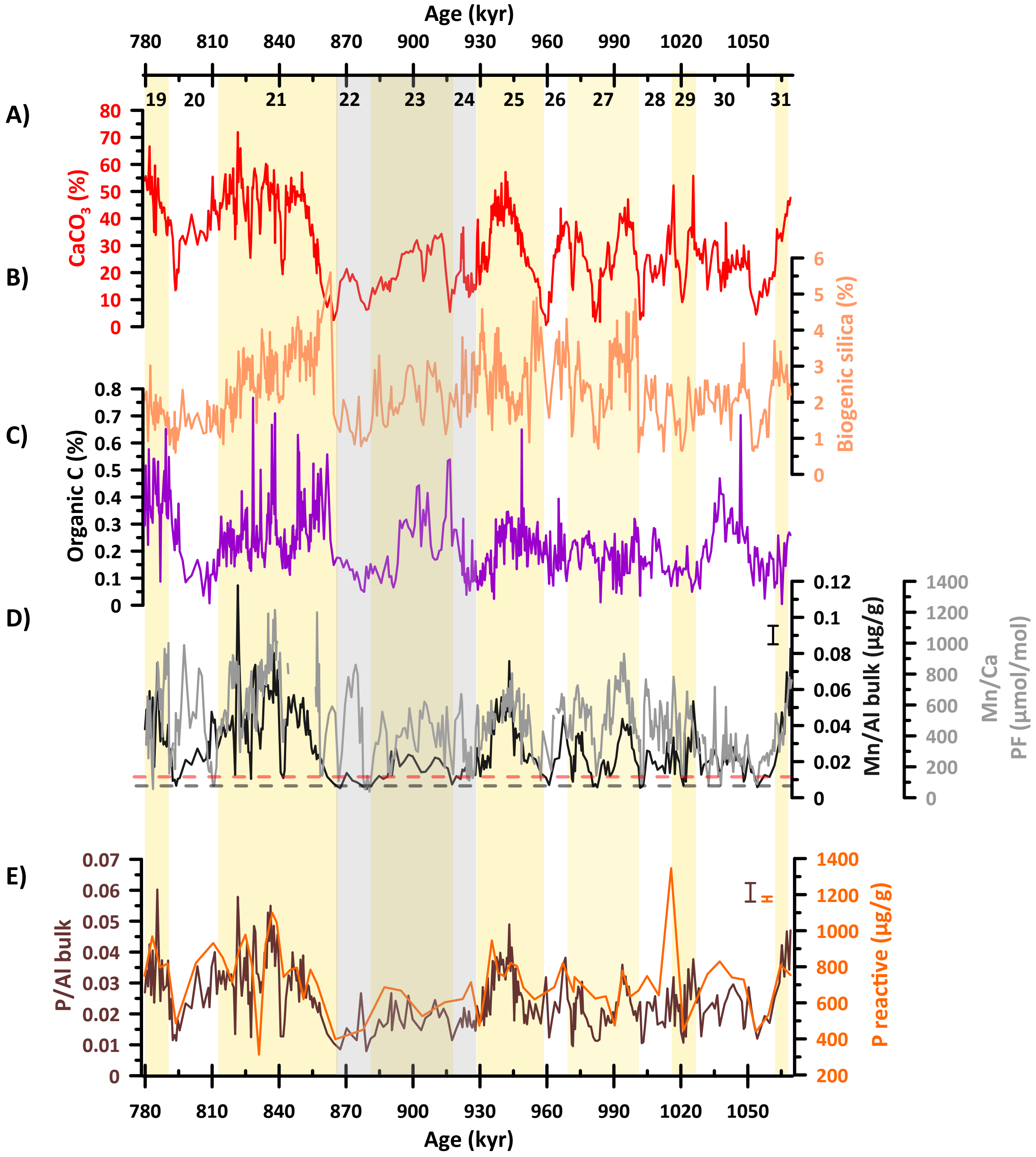

Supplementary Fig. 5. Downcore records of different geochemical proxies measured at Site U1314: (A) $CaCO_3$ (red line) [64], (B) biogenic silica (orange line) [36], (C) total organic carbon (purple line), (D) Mn/Ca ratio in planktonic foraminifera coatings (grey line) and bulk Mn/Al sediment (black line). (E) Bulk P/Al (brown line) and phosphorus reactive from sequential extraction (orange line), which includes P-Fe bound, organic, authigenic. Horizontal black dashed line indicates average upper continental crust compositions (i.e. 0.0075 for Mn/Al), and the red dashed line indicates average andesitic crust composition (i.e. 0.0116 for Mn/Al) [65]. Error bars are replicate (n=20) 1SD (see Methods). The vertical grey bar highlights MIS 24–MIS 22. Black numbers indicate interglacials (odd) and glacials (even) marine isotope stages (MIS). Yellow vertical bars correspond to interglacial stages.

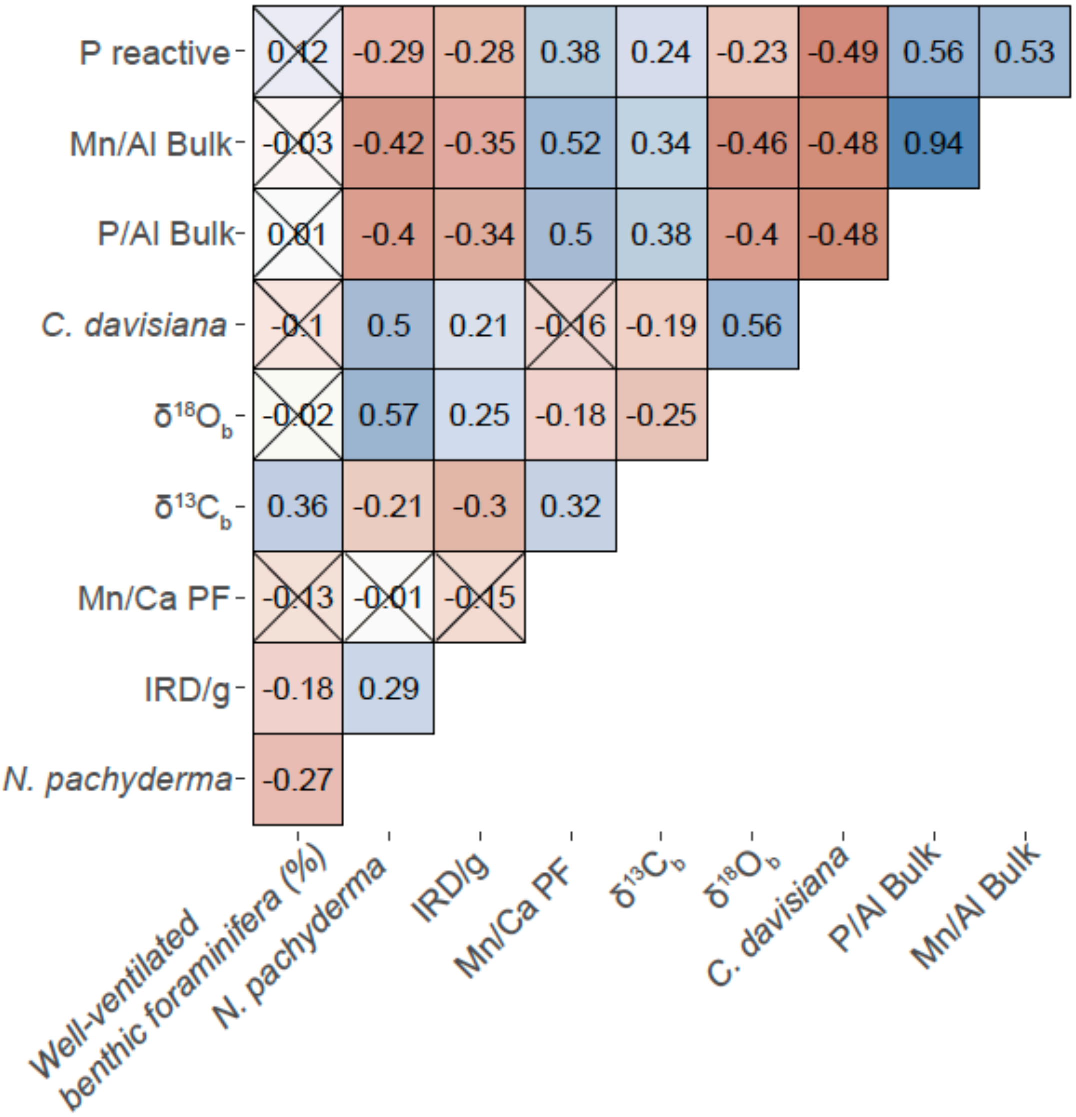

Supplementary Fig. 6. Correlation table between micropaleontological, isotopic, sedimentological, and geochemical proxies at U1314. Data interpolated at the resolution of the benthic foraminifera data (n=297). X indicates non-significant correlation (*p*= 0.05, Holm adjusted). Calculated using library 'ggplot2' as implemented in R [66].

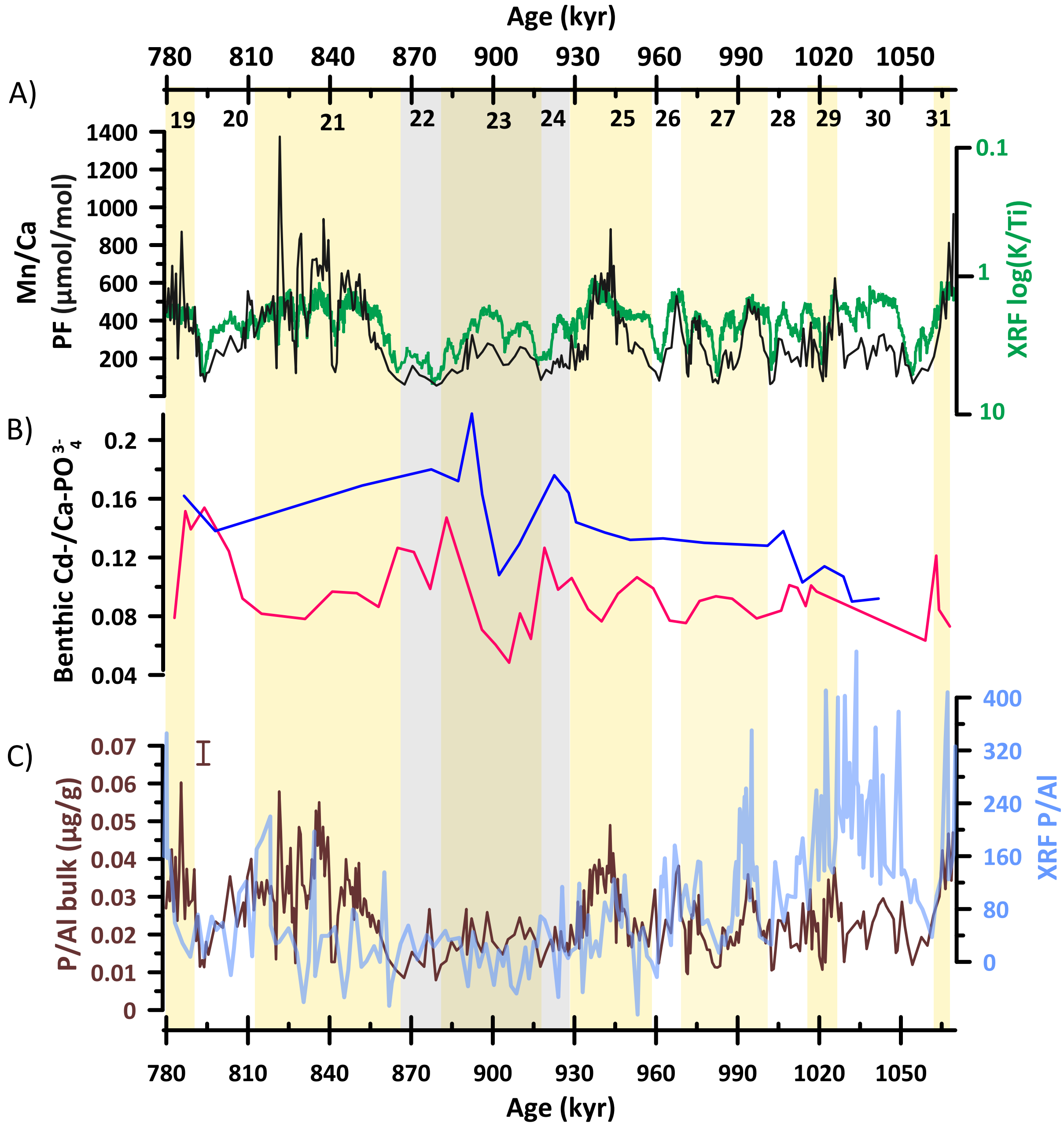

Supplementary Fig. 7. Downcore records of different geochemical proxies measured at Site U1314 and at other sites in the North Atlantic: (A) Sedimentary bulk Mn/Al (black line) and XRF log(K/Ti) [green line; 67] from site U1314. (B) Benthic foraminifera Cd/Ca-based PO_4^{3} records from sites 1267 [blue line; 68] and 607 [reoccupation of U1313, pink line; 69]. (C) Sedimentary P/Al from Site U1308 measured by core scanning XRF [70], sedimentary bulk P/Al from site U1314 analyzed by bulk sediment digestion and measured by ICP-AES (see Methods). Error bars are replicate 1SD (see Methods). The vertical grey bar highlights MIS 24–MIS 22. Black numbers indicate interglacials (odd) and glacials (even) marine isotope stages (MIS). Yellow vertical bars correspond to interglacial stages.

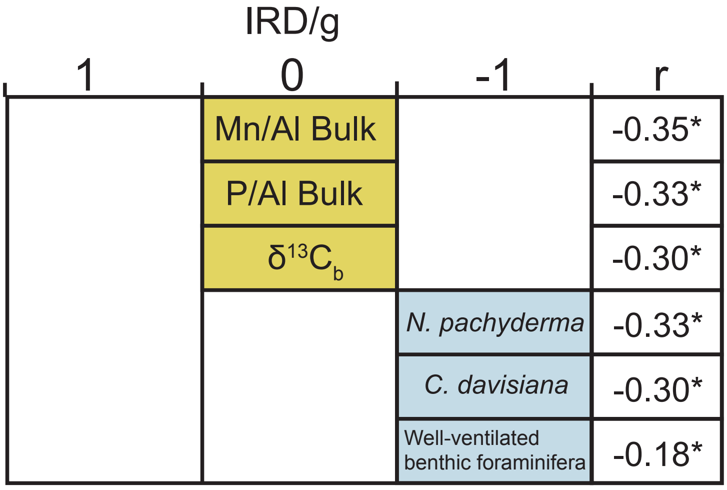

Supplementary Fig. 8. Cross-correlation between interpolated records of IRD/g and several proxies at U1314. The r value indicates the highest correlation with IRD/g at a given lag; where negative lag means that IRD leads, 0 indicates no lag and 1 that IRD follows the change of the other record. *Significant correlations at $p < 0.05$ level.

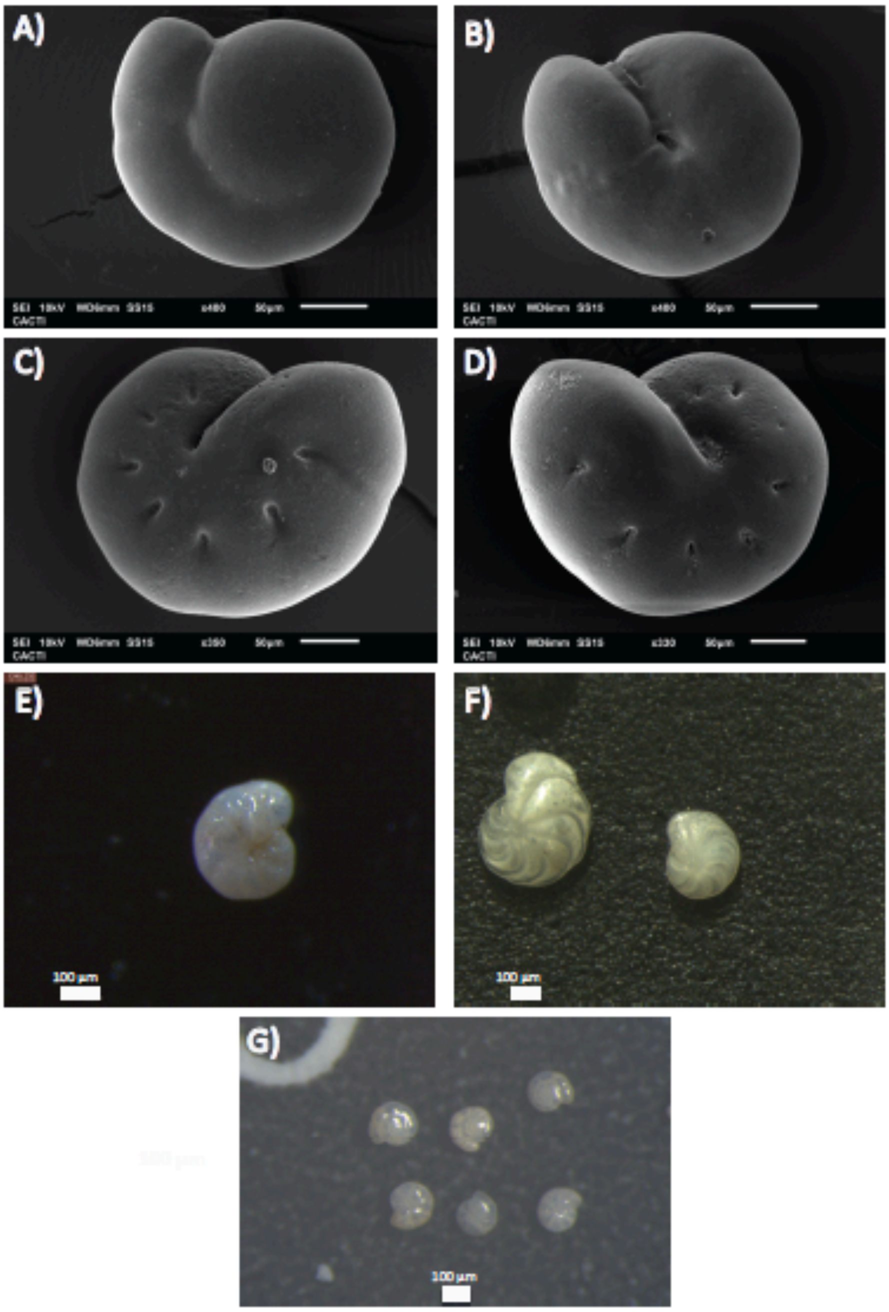

Supplementary Fig. 9. Photos of species included in the "Well-ventilated benthic foraminifera" group. (A-B) Scanning electron microscope images (SEM) of *Gyroidina umbonata,* and (C-D) *Astrononion novozealandicum.* Stereo microscope images of (E) *A. novozealandicum,* (F) *Lobatula wuellerstorfi*, and (G) *G. umbonata.*

References

1. Hernández-Almeida, I., et al., *Impact of suborbital climate changes in the North Atlantic on ice sheet dynamics at the Mid-Pleistocene Transition.* Paleoceanography, 2012. **27**(3): p. PA3214.
2. Hasenfratz, A.P., et al., *The residence time of Southern Ocean surface waters and the 100,000-year ice age cycle.* Science, 2019. **363**(6431): p. 1080-1084.
3. Jaccard, S.L., et al., *Two Modes of Change in Southern Ocean Productivity Over the Past Million Years.* Science, 2013. **339**(6126): p. 1419-1423.
4. Kanfoush, S.L., et al., *Comparison of ice-rafted debris and physical properties in ODP Site 1094 (South Atlantic) with the Vostok ice core over the last four climatic cycles.* Palaeogeography, Palaeoclimatology, Palaeoecology, 2002. **182**(3-4): p. 329-349.
5. Calvert, S.E. and T. Pedersen, *Sedimentary geochemistry of manganese; implications for the environment of formation of manganiferous black shales.* Economic Geology, 1996. **91**(1): p. 36-47.
6. Pena, L.D., et al., *Identification and removal of Mn-Mg-rich contaminant phases on foraminiferal tests: Implications for Mg/Ca past temperature reconstructions.* Geochem. Geophys. Geosyst., 2005. **6**: p. Q09P02.
7. Mangini, A., M. Jung, and S. Laukenmann, *What do we learn from peaks of uranium and of manganese in deep sea sediments?* Marine Geology, 2001. **177**(1-2): p. 63-78.
8. Boyle, E.A., *Manganese carbonate overgrowths on foraminifera tests.* Geochimica et Cosmochimica Acta, 1983. **47**(10): p. 1815-1819.
9. Boiteau, R., M. Greaves, and H. Elderfield, *Authigenic uranium in foraminiferal coatings: A proxy for ocean redox chemistry.* Paleoceanography, 2012. **27**(3).
10. Pedersen, T. and N. Price, *The geochemistry of manganese carbonate in Panama Basin sediments.* Geochimica et Cosmochimica Acta, 1982. **46**(1): p. 59-68.
11. Boyle, E. and L. Keigwin, *Comparison of Atlantic and Pacific paleochemical records for the last 215,000 years: Changes in deep ocean circulation and chemical inventories.* Earth and Planetary Science Letters, 1985. **76**(1-2): p. 135-150.
12. Yu, J., et al., *Preferential dissolution of benthic foraminiferal calcite during laboratory reductive cleaning.* Geochemistry, Geophysics, Geosystems, 2007. **8**(6).
13. Pavia, F.J., et al., *Trace metal evidence for deglacial ventilation of the abyssal Pacific and Southern Oceans.* Paleoceanography and Paleoclimatology, 2021. **36**(9): p. e2021PA004226.
14. Burdige, D.J. and J.M. Gieskes, *A pore water/solid phase diagenetic model for manganese in marine sediments.* American Journal of Science, 1983. **283**(1): p. 29-47.
15. Kasten, S., et al., *Processes and signals of nonsteady-state diagenesis in deep-sea sediments and their pore waters.* The South Atlantic in the late quaternary: Reconstruction of material budgets and current systems, 2004: p. 431-459.
16. Mangini, A., A. Eisenhauer, and P. Walter, *Response of Manganese in the Ocean to the Climatic Cycles in the Quaternary.* Paleoceanography, 1990. **5**(5): p. 811–821.
17. Koho, K., L. De Nooijer, and G. Reichart, *Combining benthic foraminiferal ecology and shell Mn/Ca to deconvolve past bottom water oxygenation and paleoproductivity.* Geochimica et Cosmochimica Acta, 2015. **165**: p. 294-306.

18. McKay, C., et al., *A comparison of benthic foraminiferal Mn/Ca and sedimentary Mn/Al as proxies of relative bottom-water oxygenation in the low-latitude NE Atlantic upwelling system.* Biogeosciences, 2015. **12**(18): p. 5415-5428.
19. Barker, S., M. Greaves, and H. Elderfield, *A study of cleaning procedures used for foraminiferal Mg/Ca paleothermometry.* Geochemistry, Geophysics, Geosystems, 2003. **4**(9).
20. Hasenfratz, A.P., et al., *Determination of the Mg/Mn ratio in foraminiferal coatings: an approach to correct Mg/Ca temperatures for Mn-rich contaminant phases.* Earth and Planetary Science Letters, 2017. **457**: p. 335-347.
21. Defforey, D. and A. Paytan, *Phosphorus cycling in marine sediments: Advances and challenges.* Chemical Geology, 2018. **477**: p. 1-11.
22. Delaney, M.L., *Phosphorus accumulation in marine sediments and the oceanic phosphorus cycle.* Global Biogeochemical Cycles, 1998. **12**(4): p. 563-572.
23. Tamburini, F., et al., *Dysaerobic conditions during Heinrich events 4 and 5: Evidence from phosphorus distribution in a North Atlantic deep-sea core.* Geochimica et Cosmochimica Acta, 2002. **66**(23): p. 4069-4083.
24. Baturin, G., *Disseminated phosphorus in oceanic sediments—A review.* Marine geology, 1988. **84**(1-2): p. 95-104.
25. Ruttenberg, K.C. and R.A. Berner, *Authigenic apatite formation and burial in sediments from non-upwelling, continental margin environments.* Geochimica et cosmochimica acta, 1993. **57**(5): p. 991-1007.
26. Ingall, E.D., R. Bustin, and P. Van Cappellen, *Influence of water column anoxia on the burial and preservation of carbon and phosphorus in marine shales.* Geochimica et Cosmochimica Acta, 1993. **57**(2): p. 303-316.
27. Ingall, E. and R. Jahnke, *Influence of water-column anoxia on the elemental fractionation of carbon and phosphorus during sediment diagenesis.* Marine Geology, 1997. **139**(1-4): p. 219-229.
28. Filippelli, G.M., *Carbon and phosphorus cycling in anoxic sediments of the Saanich Inlet, British Columbia.* Marine Geology, 2001. **174**(1-4): p. 307-321.
29. Algeo, T.J. and E. Ingall, *Sedimentary Corg: P ratios, paleocean ventilation, and Phanerozoic atmospheric pO2.* Palaeogeography, Palaeoclimatology, Palaeoecology, 2007. **256**(3-4): p. 130-155.
30. März, C., et al., *Redox sensitivity of P cycling during marine black shale formation: dynamics of sulfidic and anoxic, non-sulfidic bottom waters.* Geochimica et Cosmochimica Acta, 2008. **72**(15): p. 3703-3717.
31. Van Cappellen, P. and E.D. Ingall, *Benthic phosphorus regeneration, net primary production, and ocean anoxia: a model of the coupled marine biogeochemical cycles of carbon and phosphorus.* Paleoceanography, 1994. **9**(5): p. 677-692.
32. Filippelli, G.M. and M.L. Delaney, *Phosphorus geochemistry of equatorial Pacific sediments.* Geochimica et Cosmochimica Acta, 1996. **60**(9): p. 1479-1495.
33. Jaccard, S.L., et al., *Covariation of deep Southern Ocean oxygenation and atmospheric CO2 through the last ice age.* Nature, 2016. **530**(7589): p. 207-210.
34. Wu, L., et al., *Late Quaternary deep stratification-climate coupling in the Southern Ocean: Implications for changes in abyssal carbon storage.* Geochemistry, Geophysics, Geosystems, 2018. **19**(2): p. 379-395.
35. Wagner, M. and I.L. Hendy, *Trace metal evidence for a poorly ventilated glacial Southern Ocean.* Quaternary Science Reviews, 2017. **170**: p. 109-120.

36. Hernández-Almeida, I., et al., *A high resolution opal and radiolarian record from the subpolar North Atlantic during the Mid-Pleistocene Transition (1069–779 ka): Palaeoceanographic implications.* Palaeogeography, Palaeoclimatology, Palaeoecology, 2013a. **391, Part A**: p. 49-70.
37. Hernández-Almeida, I., et al., *Subsurface North Atlantic warming as a trigger of rapid cooling events: evidences from the Early Pleistocene (MIS 31–19).* Climate of the Past, 2015. **11**: p. 687–696.
38. Lynch-Stieglitz, J., et al., *The influence of air-sea exchange on the isotopic composition of oceanic carbon: Observations and modeling.* Global Biogeochemical Cycles, 1995. **9**(4): p. 653-665.
39. Charles, C. and R. Fairbanks, *Glacial to interglacial changes in the isotopic gradients of Southern Ocean surface water*, in *Geological History of the Polar Oceans: Arctic Versus Antarctic*. 1990, Springer. p. 519-538.
40. Spero, H., et al., *Effect of seawater carbonate concentration on foraminiferal carbon and oxygen isotopes.* Nature, 1997. **390**: p. 497-500.
41. Mackensen, A., et al., *The δ13C in benthic foraminiferal tests of Fontbotia wuellerstorfi (Schwager) relative to the δ13C of dissolved inorganic carbon in southern ocean deep water: implications for glacial ocean circulation models.* Paleoceanography, 1993. **8**(5): p. 587-610.
42. Gottschalk, J., et al., *Biological and physical controls in the Southern Ocean on past millennial-scale atmospheric CO2 changes.* Nature communications, 2016. **7**(1): p. 11539.
43. Hermelin, J.O.R., *Pliocene benthic foraminifera from the Ontong-Java plateau (western equatorial Pacific Ocean): faunal response to changing paleoenvironments.* 1989.
44. Kuhnt, W., S. Hess, and Z. Jian, *Quantitative composition of benthic foraminiferal assemblages as a proxy indicator for organic carbon flux rates in the South China Sea.* Marine Geology, 1999. **156**(1): p. 123-157.
45. Hayward, B.W., et al., *Benthic foraminiferal proxy evidence for the Neogene palaeoceanographic history of the Southwest Pacific, east of New Zealand.* Marine Geology, 2004. **205**(1-4): p. 147-184.
46. Szarek, R., H. Nomaki, and H. Kitazato, *Living deep-sea benthic foraminifera from the warm and oxygen-depleted environment of the Sulu Sea.* Deep Sea Research Part II: Topical Studies in Oceanography, 2007. **54**(1-2): p. 145-176.
47. Fillon, R.H., *Late Cenozoic foraminiferal paleoecology of the Ross Sea, Antarctica.* Micropaleontology, 1974: p. 129-151.
48. Ishman, S.E. and E.W. Domack, *Oceanographic controls on benthic foraminifers from the Bellingshausen margin of the Antarctic Peninsula.* Marine Micropaleontology, 1994. **24**(2): p. 119-155.
49. Dejardin, R., et al., *"Live"(stained) benthic foraminiferal living depths, stable isotopes, and taxonomy offshore South Georgia, Southern Ocean: implications for calcification depths.* Journal of Micropalaeontology, 2018. **37**(1): p. 25-71.
50. Majewski, W., P.J. Bart, and A.J. McGlannan, *Foraminiferal assemblages from ice-proximal paleo-settings in the Whales Deep Basin, eastern Ross Sea, Antarctica.* Palaeogeography, Palaeoclimatology, Palaeoecology, 2018. **493**: p. 64-81.
51. Corliss, B.H., *Taxonomy of Recent deep-sea benthonic foraminifera from the southeast Indian Ocean.* Micropaleontology, 1979: p. 1-19.

52. Mead, G., *Recent benthic foraminifera in the Polar Front region of the southwest Atlantic.* Micropaleontology, 1985: p. 221-248.
53. Belanger, P. and W. Berggren, *Neogene benthic foraminifera of the Hatton-Rockall Basin.* Micropaleontology, 1986: p. 324-356.
54. Nomura, R., *Paleogene to Neogene deep-sea paleoceanography in the eastern Indian Ocean: benthic foraminifera from ODP Sites 747, 757 and 758.* Micropaleontology, 1995: p. 251-290.
55. Miller, K.G. and M.E. Katz, *Oligocene to Miocene benthic foraminiferal and abyssal circulation changes in the North Atlantic.* Micropaleontology, 1987: p. 97-149.
56. Venz, K.A. and D.A. Hodell, *New evidence for changes in Plio-Pleistocene deep water circulation from Southern Ocean ODP Leg 177 Site 1090.* Palaeogeography, Palaeoclimatology, Palaeoecology, 2002. **182**(3-4): p. 197-220.
57. Hodell, D., et al., *A reference time scale for Site U1385 (Shackleton Site) on the SW Iberian Margin.* Global and Planetary Change, 2015. **133**: p. 49-64.
58. Venz, K.A., et al., *A 1.0 Myr Record of Glacial North Atlantic Intermediate Water Variability from ODP Site 982 in the Northeast Atlantic.* Paleoceanography, 1999. **14**(1): p. 42-52.
59. Ferretti, P., et al., *North Atlantic millennial-scale climate variability 910 to 790 ka and the role of the equatorial insolation forcing.* Earth and Planetary Science Letters, 2010. **293**(1-2): p. 28-41.
60. Hodell, D.A., et al., *Onset of "Hudson Strait" Heinrich events in the eastern North Atlantic at the end of the middle Pleistocene transition (~640 ka)?* Paleoceanography, 2008. **23**(4): p. PA4218.
61. Hodell, D.A. and J.E.T. Channell, *Mode transitions in Northern Hemisphere glaciation: co-evolution of millennial and orbital variability in Quaternary climate.* Climate of the Past, 2016. **12**(9): p. 1805-1828.
62. Lisiecki, E.L. and E.R. Raymo, *A Pliocene-Pleistocene stack of 57 globally distributed benthic d18O records.* Paleoceanography, 2005. **20**.
63. Railsback, L.B., et al., *An optimized scheme of lettered marine isotope substages for the last 1.0 million years, and the climatostratigraphic nature of isotope stages and substages.* Quaternary Science Reviews, 2015. **111**: p. 94-106.
64. Hernández-Almeida, I., et al., *Palaeoceanographic changes in the North Atlantic during the Mid-Pleistocene Transition (MIS 31–19) as inferred from planktonic foraminiferal and calcium carbonate records.* Boreas, 2012. **42**(1): p. 140-159.
65. Taylor, S.R. and S.M. McLennan, *The continental crust: Its Composition and Evolution*. 1985, Malden, Massachusetts: Blackwell. 328.
66. R Core Team, *R: A language and environment for statistical computing. R Foundation for Statistical Computing, Vienna, Austria.* 2018.
67. Gruetzner, J. and S. Higgins, *Threshold behavior of millennial scale variability in deep water hydrography inferred from a 1.1 Ma long record of sediment provenance at the southern Gardar Drift.* Paleoceanography, 2010. **25**(4).
68. Farmer, J., et al., *Deep Atlantic Ocean carbon storage and the rise of 100,000-year glacial cycles.* Nature Geoscience, 2019. **12**(5): p. 355-360.
69. Lear, C.H., et al., *Breathing more deeply: Deep ocean carbon storage during the mid-Pleistocene climate transition.* Geology, 2016. **44**(12): p. 1035-1038.

70. Thomas, N.C., H.J. Bradbury, and D.A. Hodell, *Changes in North Atlantic deep-water oxygenation across the Middle Pleistocene Transition.* Science, 2022. **377**(6606): p. 654-659.
