# Supplementary material for: Glacial dysoxia in the deep subpolar North Atlantic during the Mid-Pleistocene Transition
Source: Nat Commun. 2026 Apr 24;17:3748. doi: 10.1038/s41467-026-71268-4 (PMC13109349; doi:10.1038/s41467-026-71268-4)
Supplement: Supplementary file 2 — Description of Additional Supplementary Files [file 41467_2026_71268_MOESM2_ESM.pdf]

Description of Additional Supplementary Files

File name: Supplementary Data 1
Description: Bulk sediment geochemistry IODP Site 306-U1314

File name: Supplementary Data 2
Description: Sedimentary phosphorus components from IODP Site 306-U1314

File name: Supplementary Data 3
Description: Benthic foraminifera counts of key species from IODP Site 306-U1314

File name: Supplementary Data 4
Description: Organic carbon content from IODP Site 306-U1314
